# Cutaneous metastases from primary solid and hematopoietic neoplasms at a referral hospital in Colombia: a cross-sectional study^☆^^☆☆^

**DOI:** 10.1016/j.abd.2019.12.005

**Published:** 2020-05-11

**Authors:** María Fernanda Ordóñez-Rubiano, Victoria Lucía Dávila-Osorio, Paula Celeste Rubiano-Mojica, Ángela Marcela Mariño-Álvarez

**Affiliations:** a Department of Dermatology, Central Military Hospital of Bogotá, Bogotá, Colombia; b Department of General Medicine, Universidad del Rosario, Bogotá, Colombia

Dear Editor,

Cutaneous metastases (CM) represent 2% of all skin tumors and they are present in up to 10% of all cancer patients. Their clinical and histopathological manifestations are variable and depend on the primary tumor, age, and sex.^1^

Currently in Colombia there are no CM studies registered, therefore this is a pioneering study in the country.

An observational, retrospective, cross-sectional study was conducted. Medical records were reviewed from patients with histopathologically confirmed CM at the Central Military Hospital in Bogotá, from January 2015 to June 2018. Patients with skin primary tumors and those without follow-up records of at least six months were excluded from the analysis. A database was built in Microsoft Excel® including clinical and histopathological features of the primary tumor and metastases. Subsequently, a descriptive statistical analysis was carried out with the software SPSS v. 20®.

A total of 26 CM cases were collected, but five patients were excluded from the analysis due to the presence of primary tumors that originated in the skin. The average age was 56 years and 52.38% (*n* = 11) were males. The primary tumors most frequently associated were those originating in the breast (28.5%), followed by the bone marrow (23.8%).

The average time between symptoms onset and diagnosis of CM was 2.84 months, and the mean number of skin lesions was 3.76. The most frequent locations were chest 28.5% (*n* = 6) and abdomen (19%), and regarding clinical presentation, nodules 52.3% (*n* = 11) were the most commonly seen. CM behavior was evaluated with different immunohistochemical markers, whereupon three patients with elevated Ki-67 in the CM tissue compared to the primary tumor were identified (Table 1).

**Table 1:** Clinical and histopathological correlation of cutaneous metastases.

Primary tumor	Frequency	Gender		Age in years (m)	Primary histological diagnosis	Metastasis histological diagnosis	Immunohistochemistry	Skin location	Type of lesion
		F	M						
Breast	28.5%	100%	0%	57.3	Ductal adenocarcinoma (50%)	Adeno-carcinoma	Estrogen loss: one Progesterone gain: one CK-20 gain: one Ki-67, two patients: 10–89% and 5–30%	Chest, scalp	Nodules, plaques
Kidney	9.5%	0%	100%	53.5	Clear cell renal cell carcinoma (100%)	Metastatic clear cell renal cell carcinoma	No changes	Scalp, finger	Nodules, pedunculated tumor
Lung	4.7%	100%	0%	74	Adenocarcinoma	Adenocarcinoma	No changes	Abdomen	Nodule
Bone marrow	23.8%	25%	75%	50.4	ALL (50%), AML (50%)	Leukemia cutis, leukemid	No changes	Face, abdomen, perianal area	Nodules, ulcers, plaques
Muscle	4.7%	0%	100%	19	Epitheloid sarcoma	Epitheloid sarcoma	No changes	Face	Ulcerated nodule
Rectum	4.7%	0%	100%	69	Adenocarcinoma	Adenocarcinoma	No changes	Superior lip	Nodule
Parotid	4.7%	0%	100%	73	Mucoepidermoid carcinoma	Mucoepidermoid carcinoma	Her – 2 loss	Scalp	Nodules
Brain	4.7%	0%	100%	44	Glioblastoma	Glioblastoma	Ki-67: 20–40%	Scalp	Plaques
Lymphatic	4.7%	100%	0%	61	Follicular lymphoma	B cell lymphoma	No changes	Abdomen	Plaque
Unknown	9.5%	50%	50%	66.5	Does not apply	Adeno-carcinoma	–	Bilateral inguinal	Tumor, nodules

ALL, acute lymphoblastic leukemia; AML, acute myeloid leukemia.

Relapse of the primary tumor before the appearance of CM was observed in 47.6% of the patients. The majority of patients (80.9%) received systemic chemotherapy, with a skin response only in 23.8% (*n* = 5) of all cases. Only four had an intervention for the CM (radiotherapy: three; surgery: one). The survival time after the skin diagnosis was 10.65 months.

CM are defined as a dissemination of malignant cells from a primary malignancy toward the skin, compromising the epidermis, dermis, or hypodermis.^1^ It occurs in up to 10.4% of all patients with cancer and represents 2% of all skin tumors.^1^

Despite being an uncommon entity in daily practice, they have an important clinical significance because they usually indicate advanced disease, as in the present study.^2^

In the majority of cases, the most frequently primary tumor associated with CM in women is breast cancer, while in men, excluding melanoma, it is lung cancer; nevertheless, the present study did not identify any male patients with CM from lung cancer.^1^, ^3^

In general, carcinomas are the most common metastasis-producing type of cancer but, as shown in this report, CM originate frequently in adenocarcinomas.^4^

Clinically, they present a wide variety of manifestations, such as nodules, papules, plaques, tumors, and ulcers, sometimes are associated to pain, and have a tendency to affect the scalp, trunk, and neck.^2^

Some morphological patterns have been defined histopathologically, and from time to time these may resemble findings of the primary malignancy, in which immunochemistry plays a fundamental role.^1^, ^5^

Finally, early recognition of CM has an important effect on patient prognosis, especially in those with a primary tumor of unknown origin, where histopathology can guide the diagnosis, or in patients with a recurrent tumor, where it could alert to an active cancer.

## Financial support

None declared.

## Authors’ contributions

Ordóñez Rubiano María Fernanda: Statistical analysis; approval of the final version of the manuscript; conception and planning of the study; drafting and editing of the manuscript; collection, analysis, and interpretation of data; participation in study design; intellectual participation in the propaedeutic and/or therapeutic conduct of the studied cases; critical review of the literature; critical review of the manuscript.

Dávila Osorio Victoria Lucía: Statistical analysis; approval of the final version of the manuscript; conception and planning of the study; drafting and editing of the manuscript; collection, analysis, and interpretation of data; participation in study design; intellectual participation in the propaedeutic and/or therapeutic conduct of the studied cases; critical review of the literature; critical review of the manuscript.

Rubiano Mojica Paula Celeste: Approval of the final version of the manuscript; drafting and editing of the manuscript; critical review of the literature; critical review of the manuscript.

Mariño Álvarez Ángela Marcela: Statistical analysis; approval of the final version of the manuscript; conception and planning of the study; drafting and editing of the manuscript; collection, analysis, and interpretation of data; participation in study design; intellectual participation in the propaedeutic and/or therapeutic conduct of the studied cases; critical review of the literature; critical review of the manuscript.

## Conflicts of interest

None declared.
